# Brain transcriptome of the violet-eared waxbill *Uraeginthus granatina* and recent evolution in the songbird genome

**DOI:** 10.1098/rsob.130063

**Published:** 2013-09

**Authors:** Christopher N. Balakrishnan, Charles Chapus, Michael S. Brewer, David F. Clayton

**Affiliations:** 1 Department of Biology, East Carolina University, Greenville, NC 27858, USA; 2 Institute for Genomic Biology, University of Illinois at Urbana–Champaign, Urbana, IL 61801, USA; 3 UMR-MD3, Institut de Recherche Biomédicale des Armées, Antenne Marseille, Marseille, 13007, France; 4 Department of Environmental Science, Policy and Management, University of California, Berkeley, CA 94720, USA

**Keywords:** zebra finch, genome, positive selection, sex chromosome, aggression, social behaviour

## Abstract

Songbirds are important models for the study of social behaviour and communication. To complement the recent genome sequencing of the domesticated zebra finch, we sequenced the brain transcriptome of a closely related songbird species, the violet-eared waxbill (*Uraeginthus granatina*)*.* Both the zebra finch and violet-eared waxbill are members of the family Estrildidae, but differ markedly in their social behaviour. Using Roche 454 RNA sequencing, we generated an assembly and annotation of 11 084 waxbill orthologues of 17 475 zebra finch genes (64%), with an average transcript length of 1555 bp. We also identified 5985 single nucleotide polymorphisms (SNPs) of potential utility for future population genomic studies. Comparing the two species, we found evidence for rapid protein evolution (*ω*) and low polymorphism of the avian Z sex chromosome, consistent with prior studies of more divergent avian species. An intriguing outlier was putative chromosome 4A, which showed a high density of SNPs and low evolutionary rate relative to other chromosomes. Genome-wide *ω* was identical in zebra finch and violet-eared waxbill lineages, suggesting a similar demographic history with efficient purifying natural selection. Further comparisons of these and other estrildid finches may provide insights into the evolutionary neurogenomics of social behaviour.

## 2. Introduction

To date, nearly 50 mammalian genomes have been completely sequenced. These diverse genome sequences capture many of the major lineages in the mammalian tree of life, and frame the study of evolution on a broad scale. Detailed sampling of closely related species, however, provides a complementary perspective. Analyses of closely related primate species have begun to reveal the molecular [1,2] and regulatory [3] changes underlying species differences. Similarly, among insects the effort to sequence 12 *Drosophila* species has yielded insights into patterns of nucleotide and gene family evolution [4–6].

Of over 10 000 species in the avian radiation, seven genomes have now been sequenced to near completion [7–12]. Two of these, the chicken (*Gallus gallus*) and the turkey (*Melagris gallopavo*), represent a single avian family (Phasianidae). The Phasianidae include a number of species of importance in the food industry, and the chicken in particular has been important in studies of developmental biology and immunology. Falcon (Falconidae), pigeon (Columbidae) and mallard duck (Anatidae) genomes have also recently been sequenced [10–12]. Passerines are the most diverse avian order, including over half of all bird species. Within this group, the only published genome is that of the zebra finch *Taeniopygia guttata* [8]. The zebra finch was selected for sequencing as a representative of the passerines, and specifically due to its prominent role as an experimental system for the study of neurobiology and behaviour [13–15].

Owing to the limited genomic data currently available for birds, studies of avian molecular evolution have largely been restricted to comparisons between the zebra finch and chicken [8,16,17] (but see [9,12]). Zebra finch and chicken lineages diverged between 75 and 150 Ma, thus bracketing all of avian diversity with the exception of the palaeognathes [18–20]. Comparisons of zebra finch and chicken therefore necessarily average evolutionary signatures across much of avian diversity. In doing so, the signal of recent adaptation in songbirds has been obscured [17]. Next-generation sequencing studies are driving a rapid increase in the genome-scale data available for birds [21,22], but these datasets too have targeted divergent taxa. Transcriptome datasets, however, offer the potential to rapidly fill in the avian tree of life [21,23–27], allowing comparisons of closely related taxa towards the understanding of molecular evolution on more recent time scales.

Birds in general display striking variation in social behaviour [28], and the estrildid finches (family Estrildidae, 143 species) in particular have been cited as important model systems for studying the behavioural, endocrinological and neural substrates of sociality [29–31]. Within the estrildids, the zebra finch in some ways represents one extreme along a continuum of variation in social behaviour. Zebra finches are highly colonial, living in large, nomadic flocks in Australia and southeast Asia. The other extreme is represented by the violet-eared waxbill (*Uraeginthus granatina*), native to Africa. In contrast to the zebra finch and many other estrildids, the waxbill is highly territorial, especially during the breeding season. These two species diverged around 10–15 Ma at the base of the estrildid finch radiation [32,33]. Notable progress has been made in defining neurobiological elements underlying vocal and social communication (especially in the zebra finch [34–40]) and territoriality (especially in the violet-eared waxbill [31]).

Thus, the violet-eared waxbill is both an emerging model organism in its own right, and an intriguing phylogenetic contrast to the zebra finch for studies of avian evolution and sociality. With this in mind, we sequenced the brain transcriptome of the violet-eared waxbill. We present here a detailed picture of transcription in the waxbill brain and molecular evolution in recently diverged songbird lineages, taking additional advantage of the recent publication of the transcriptome of a non-estrildid songbird species, the great tit (*Parus major*) [23].

## 3. Material and methods

### 3.1. 454 Library preparation sequencing and assembly

RNA was extracted from whole brain tissue (provided by Jim Goodson, Indiana University) of a male violet-eared waxbill that had been snap-frozen on dry ice. The frozen whole brain was manually fragmented and homogenized in Tri-reagent (Ambion). RNA was then extracted from the homogenate following the manufacturer's protocol. Purified RNA was checked for quality using an Agilent Biolanalyzer. Fifty micrograms of total RNA were further purified using a Qiagen RNeasy spin column to further remove any possible DNA contamination. Polyadenylated RNA was selected from the total RNA with an Oligotex mini kit (Qiagen). Poly-A-selected RNA was then reverse transcribed using random primers. cDNA was fragmented using a nebulizer to generate fragments ranging from approximately 400 to 800 bp in length. We then normalized the cDNA library using the Trimmer Direct kit (Evrogen) and manufacturer's protocols. End repair, 3′ addition of A bases and ligation of adaptors were done following Illumina library preparation guidelines but using Roche 454 adaptors. We then ran the library in agarose and gel purified the 400–800 bp band.

Sequences were assembled using gsAssembler software (v. 2.3) from Roche. Reads that contained homopolymers (60% over the entire length of the read represented by one nucleotide) and reads that were shorter than 100 bp were filtered. The parameters used for the assembly were overlapMinMatchLength of 40 and overlapMinMatchIdentity of 90%. The resulting isotigs and singlets that were more than 100 bp were annotated using BLAST against non-redundant protein database from NCBI (http://www.ncbi.nlm.nih.gov), chicken proteins, and zebra finch transcripts, proteins and genome from Ensembl (http://uswest.ensembl.org/).

### 3.2. Single nucleotide polymorphism detection and analysis

We used DIAL (De novo Identification of Alleles [41]) to identify single nucleotide polymorphisms (SNPs) in the waxbill transcriptome. DIAL is specifically tailored to identify SNPs in species for which a reference genome is lacking. DIAL incorporates platform-specific pipelines (in this case, we specified Roche 454 sequencing) and a specific algorithm for cDNA sequencing (-transcript flag). Because we sequenced a single individual, our discovered waxbill SNPs are due to heterozygosity and are biased towards the identification of high-frequency polymorphisms. The approach in DIAL uses Newbler, the Roche assembly algorithm, to construct clusters within which polymorphisms are surveyed. SNPs discovered by DIAL were then mapped to the zebra finch genome. We assumed conservation of genome structure between waxbill and zebra finch to assess chromosomal location of waxbill SNPs and their location relative to genes (intergenic, intronic and genic). This assumption is reasonable given the broad conservation of synteny across birds [8,42]. Cytogenetic analyses (c- and g-banding) of estrildid finch chromosomes have also been conducted, revealing structural polymorphisms within the group [43]. These previous analyses, however, emphasized intrachromosomal polymorphisms, which are common, but would not impact the conclusions drawn here.

### 3.3. Sequence alignment and molecular rate analyses

To align sequences from the new violet-eared waxbill transcriptome, we compared assembled transcripts with annotated zebra finch cDNAs and with another recently published songbird transcriptome—that of the great tit [23]—using BLAST+2.2.25. The whole set of waxbill and great tit transcripts were compared with the zebra finch database using the following parameters: expected e-value = 1 × 10^−5^ and minimum length of the BLAST hit = 300 bp. For each waxbill and great tit transcript, we kept the zebra finch hit satisfying these criteria and with the best e-value.

In order to conduct downstream molecular rate analyses, we refined alignments to match homologous codons across species and remove any non-coding regions. To refine alignments, we used MUSCLE [44,45] with the -diags parameter invoked to improve alignment speed given the similarity of the species in question. We mapped the MUSCLE alignments onto the Ensembl zebra finch cDNA using the starting coordinate of the previously derived BLAST alignments. Where there were extra nucleotides in the transcriptome sequences (waxbill or great tit) that disrupted open reading frames, we treated them as sequencing errors and discarded the extra bases. Ensembl-annotated zebra finch cDNAs by definition all maintain an open reading frame, and we sought to maintain these annotated gene structures in our alignments. For waxbill and great tit, where multiple isoforms may have been assembled, this approach preferentially selects the most similar isoform to that represented in the zebra finch genome assembly. It is possible that different isoforms are present in the transcriptome(s) than in the Ensembl annotation, but in this case, exons not represented in the zebra finch gene models will simply not be represented in the analysis.

In many cases, there was more than one waxbill and/or great tit assembled transcripts mapped to each zebra finch cDNA. This was because individual isotigs often did not span the entire length of the zebra finch gene. We therefore determined a consensus sequence of each gene for each species. We trimmed the alignments by using the zebra finch CDS positions as a guide, and removed terminal stop codons for each sequence in the alignments. We used the same general approach to generate pairwise alignments (zebra finch–waxbill, zebra finch–great tit, great tit–waxbill). As these alignments required only two species to overlap, they resulted in a larger number of alignments and longer alignments. The quality and method of sequence alignment have important impacts on inferences regarding rates of evolution [46]. During the course of this study, we tested multiple alignment pipelines, including the use of amino acid sequence-based approaches [47]. Visual inspection of alignments supported the use of our MUSCLE-based pipeline.

We analysed molecular evolutionary patterns using PAML (phylogenetic analysis using maximum likelihood) [48,49]. For pairwise alignments, we used the pairwise *ω* (d*N*/d*S*) estimators implemented in codeml. We also used the likelihood approach in codeml and tested ‘branch’ models in which waxbill or zebra finch was allowed to have an independent rate relative to a null model where all three species had a fixed rate. We then used likelihood ratio tests to identify genes whose rate was significantly accelerated in either the waxbill or the zebra finch lineage. *p*-values were determined using a χ^2^-distribution with one degree of freedom and were adjusted for multiple testing (*q* < 0.05) using *Q*-value [50]. To examine molecular rates at different time scales, we also compared rates from zebra finch/waxbill codeml comparisons with those from zebra finch/chicken comparisons. Estimates of *ω* from zebra finch relative to chicken were made using Ensembl Biomart (ensemble.org).

To estimate the overall rate of protein evolution (*ω*) across the genome, we generated bootstrap datasets based on our previous alignment following Heger & Ponting [5]. For each species pair, we generated 1000 bootstrap datasets by concatenating 150 randomly chosen alignments. These longer alignments generate more robust and reliable estimates of genome-wide *ω* [5]. Across each of the 1000 alignments, we compared average *ω* between pairwise analyses of zebra finch–great tit and waxbill–great tit to test for rate variation between these two species, as might be expected if the two species had different demographic histories.

Gene lists (genes represented in the transcriptome assembly, genes showing rate variation) were functionally described using gene ontology (GO) analyses using CORNA [51] as implemented in a public web server (www.ark-genomics.org/tools/GOfinch). Fisher's exact tests and hypergeometric tests were conducted to test for statistical over- and under-representation of GO terms. All statistics were adjusted for multiple hypotheses testing using the method of Benjamini & Hochberg [52], and *p*-values given below are adjusted *p*-values unless otherwise noted.

## 4. Results

### 4.1. Assembly and annotation

**Figure 1.**
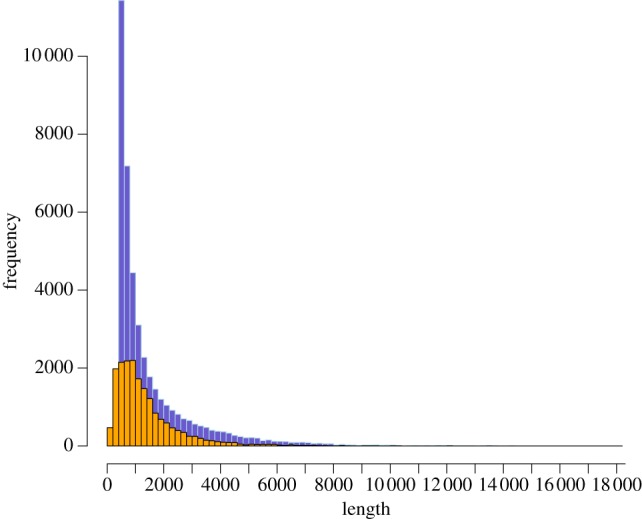
Distribution of 454 isotig size of violet-eared waxbill (purple, *n* = 43 616) versus Ensembl cDNA sequences (orange, *n* = 18 597).

Two plates of 454 sequencing yielded 975 606 and 1 055 860, reads with average read lengths of 323.5 bp and 380.5 bp, respectively. Raw sequence data have been deposited at the NCBI short read archive under accession no. SRX337999. The total waxbill brain transcriptome dataset therefore consisted of 2 031 466 reads, and 2 013 275 after filtering. Transcriptome assembly yielded 32 938 isogroups (genes) and 43 137 isotigs (transcripts) with an average size of 1555 bp and an N50 value of 2486 bp. This compares favourably with recent de novo transcriptome assemblies of the zebra finch (mean contig length = 150 bp [53]), great tit (mean contig length = 871 bp [23]) kiwi (mean contig length = 162 bp [26]) and other bird species [21]. The transcript set described here closely matches the transcript length profile for the full Ensembl transcript set for zebra finch (figure 1). The longest assembled isotig in our assembly spanned 17 589 bp, covering the primary transcript of the mitochondrial genome. Four hundred and seventy nine contigs could not be placed in isotigs, but were included in subsequent analyses, yielding 43 616 putative transcripts. A total of 233 903 singleton reads were not included in the assembly. BLAST analysis of assembled transcripts found significant matches to 7817 Ensembl genes out of 17 475 total zebra finch Ensembl genes (44.7%). Including singletons in our count of detected transcripts expands our transcriptome coverage to 11 084 Ensembl genes (63.4%).

### 4.2. Gene functional representation

**Figure 2.**
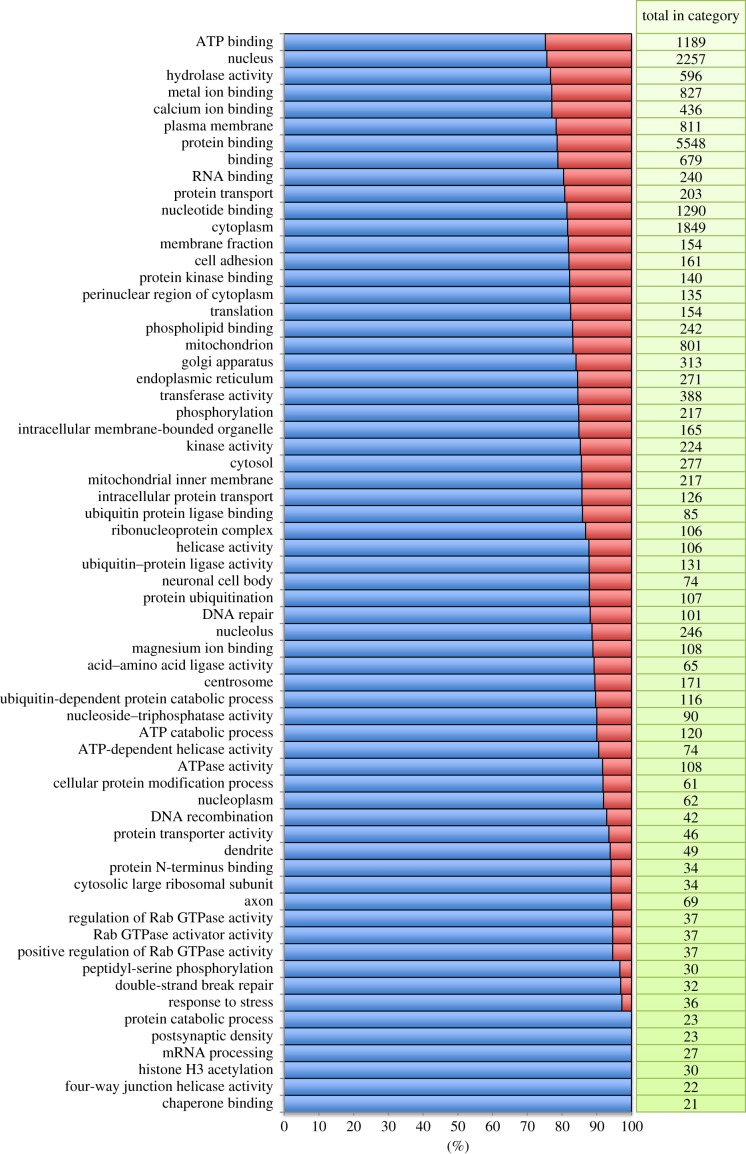
Significantly over-represented GO terms (Fisher's exact test FDR < 0.05) in the violet-eared waxbill brain transcriptome. The blue portion of each column represents the proportion of the total number genes within each GO category based on zebra finch that was detected in the waxbill assembly. The red portion of each bar represents the proportion that was not detected.

**Figure 3.**
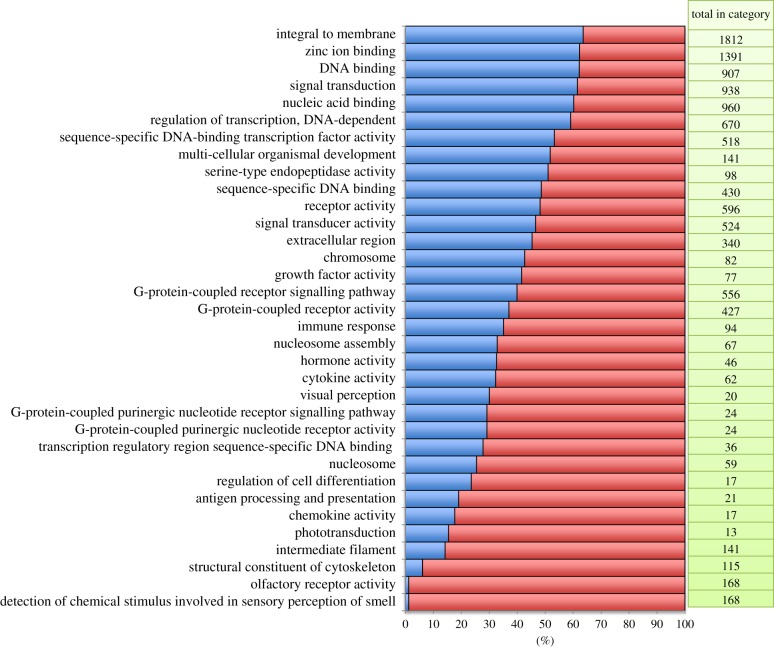
Significantly under-represented GO terms (Fisher's exact test FDR < 0.05) in the violet-eared waxbill brain transcriptome. The blue portion of each column represents the proportion of the total number genes within each GO category based on zebra finch that was detected in the waxbill assembly. The red portion of each bar represents the proportion that was not detected.

We detected a total of 7027 GO categories in our dataset of which a number were significantly over- and under-represented (Fisher's exact test) relative to the full zebra finch Ensembl gene set (figures 2 and 3; electronic supplementary material, table S1). Terms describing cellular components including endoplasmic reticulum (*p* = 1.3 × 10^−6^), cytoplasm (*p* = 8.3 × 10^−36^) and cytosol (*p* = 8.4 × 10^−8^) were significantly enriched. As might be expected of a brain-derived library, categories of neurobiological function were also enriched (axon, *p* = 0.0001; dendrite, *p* = 0.0048; neuronal cell body, *p* = 0.019). A broad group of categories related to RNA processing and translation were also enriched. Under-represented categories were particularly intriguing and included categories related to transcriptional regulation (e.g. regulation of transcription, DNA-dependent, *p* = 3.8 × 10^−6^). Also lacking were immune-related transcripts (e.g. immune response, *p* = 3.7 × 10^−9^) and olfactory receptors (ORs; *p* = 2.5 × 10^−79^). Only two OR genes were detected relative to an expectation of 116 and total of 168 annotated ORs in the zebra finch genome (see also the electronic supplementary material, table S1).

### 4.3. Distribution of genetic variation in the transcriptome

Heterozygosity of the sequenced waxbill individual allowed us to identify SNPs in the transcriptome read data. Using DIAL, we identified 5985 SNPs, of which 5641 (94.3%) could be mapped to the zebra finch genome. Our transcriptome includes reads that map to known genes and reads that map outside of them, including both putatively intergenic and intronic compartments. Reads mapping outside of Ensembl-annotated genes may represent novel, previously undescribed transcripts. Reads mapping to annotated introns may represent alternative or incomplete splicing (precursor mRNA). A total of 22.9% of SNPs mapped to known coding sequences, and another 12.9% mapped to within 1 kb of the 3′ flanking end of gene annotations. A total of 38.4% of the SNPs mapped intergenically (not including those in the ‘flanking’ category above) and 22.0% mapped to annotated introns. The remaining reads mapped annotated UTRs, telomeres and the 5′ flanking region (within 1 kb) of known genes.

**Figure 4.**
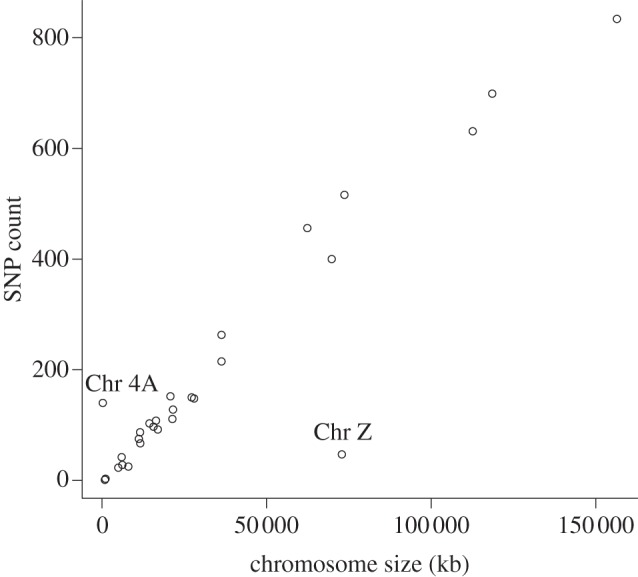
Distribution of SNPs among zebra finch chromosomes. In general, the number of discovered SNPs scales with chromosome size. Two notable exceptions, however, are the Z sex chromosome and chromosome 4A.

SNPs mapped to 28 zebra finch chromosomes and the number of SNPs detected per chromosome scaled linearly with chromosome size (figure 4). One striking exception to this pattern was the sex chromosome Z, which showed a marked reduction in SNP density. By contrast, chromosome 4A showed a much higher density of SNPs than any of the other chromosomes to which we mapped SNPs. Across all chromosomes, the SNPs were distributed at a density of 0.02 SNPs/kb, whereas chromosome 4A had 375 SNPs on an assembled chromosome of only 258 280 bp (0.54 SNPs/kb).

### 4.4. Patterns of gene and genome evolution

#### 4.4.1. Pairwise comparisons between zebra finch and violet-eared waxbill

We generated alignments for 5310 putative orthologues for zebra finch and violet-eared waxbill. This gene set was restricted to alignments of 300 bp or greater, and averaged 1367 bp in length after removal of gapped and ambiguous positions. Of these alignments, 63 (0.01%) had *ω* > 1, the traditional benchmark for adaptive evolution [54]. This list was significantly enriched for 13 GO categories (*p* = 0.05; see electronic supplementary material, table S1), but these tended to be small GO categories where the null expectation was 0 and the observation was one gene. Four categories pertaining to transcriptional regulation were enriched but fell short of statistical significance after correction for multiple testing (sequence-specific DNA binding, *p* = 0.083; regulation of transcription, DNA-dependent, adjusted *p* = 0.11; transcription factor activity, *p* = 0.14). These categories were represented by three, four and three genes, respectively.

To more broadly describe rate variation among genes, we also examined gene lists of *ω* > 0.8 (112 genes) and the overall top 10% most rapidly evolving genes (530 genes; *ω* > 0.4075). At *ω* > 0.8, three GO categories (spindle astral microtubule organization, interkinetic nuclear migration and regulation of microtubule-based process) were significant (*p* < 0.05). GO categories neurogenesis, NADH dehydrogenase activity and cerebral cortex development bordered on statistical significance (*p* = 0.075). Among the top 10%, no GO categories were significant after correction for multiple comparisons. Four genes annotated as having cytokine receptor activity (out of six total such genes in the dataset) were in the top 10% of fastest-evolving genes, and this category also bordered on statistical significance (*p* = 0.12). Several functional categories were also moderately under-represented, suggesting stabilizing selection on the genes comprising these categories (protein serine/threonine kinase activity, protein tyrosine kinase activity, protein amino acid phosphorylation and protein kinase activity, 0.05 < *p* < 0.15).

**Figure 5.**
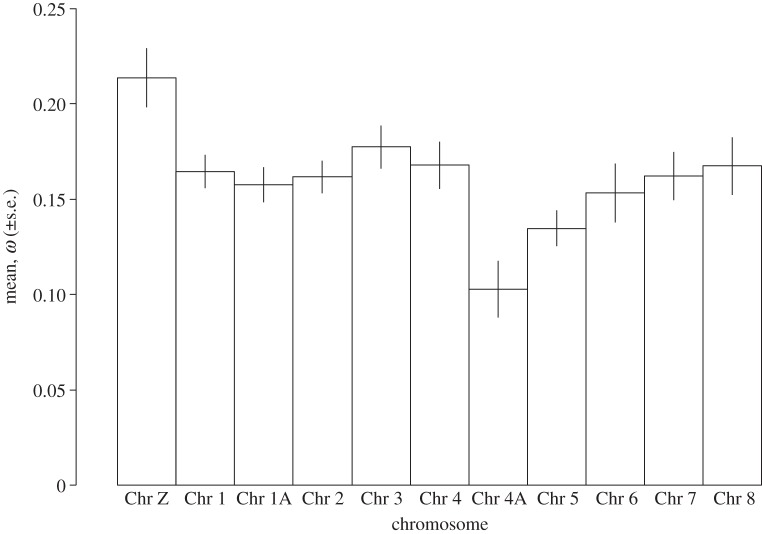
Pairwise *ω* across chromosomes 1–8 and Z (the macrochromosomes). There was significant variation among chromosomes in *ω*, with chromosomes Z and 4A showing a significant increase and decrease, respectively, from the other chromosomes.

Pairwise *ω* estimates also revealed variation of molecular evolutionary rate by chromosome (ANOVA, *p* = 0.0002; figure 5). Genes of the Z sex chromosome are evolving faster than the other chromosomes (figure 4), and significantly faster than chromosome 4 (one-tailed *t*-test, *p* = 0.01), the chromosome closest to Z in the number of aligned genes (Chr Z = 326 genes and Chr 4 = 364 genes). Chromosome 4A is also evolving slowly relative to chromosome 4, from which it is derived (one-tailed *t*-test, *p* = 0.005), and chromosome 12, the chromosome most similar in gene number (one-tailed *t*-test, *p* = 0.009).

**Figure 6.**
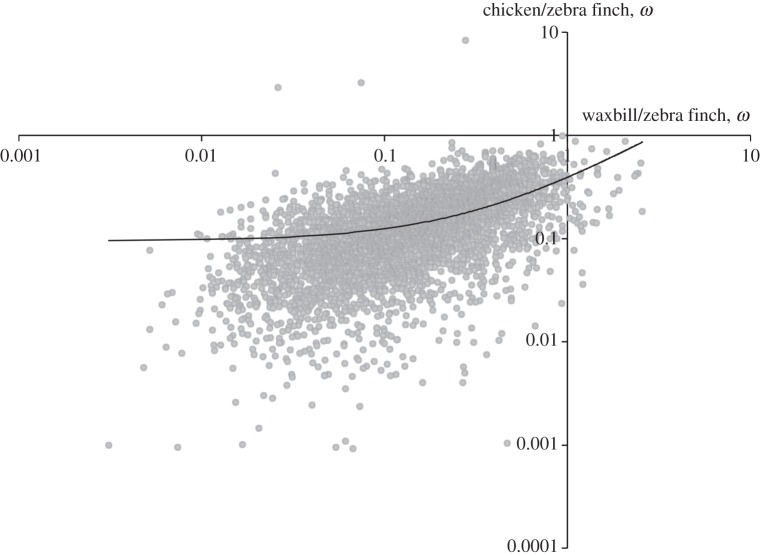
Significant correlation (*r* = *R* = 0.35, *p* < 0.001) in *ω* derived from chicken/zebra finch orthologues (Ensembl Biomart) versus those from zebra finch/waxbill orthologues (this study).

To estimate rates of evolution across the genome as a whole, we generated bootstrap datasets (see Material and methods) from pairwise alignments of each of our ingroup taxa (zebra finch and violet-eared waxbill) with an outgroup, the great tit. In each case, we estimated the average genomic *ω* between to be 0.13. Therefore, we found no difference in overall molecular rate between the two estrildid finch lineages. We also found a significant correlation between *ω* estimated from zebra finch/chicken orthologues in Ensembl and zebra finch/waxbill orthologues estimated here (*R* = 0.35, *p* < 0.001; figure 6), supporting broadly similar patterns of molecular evolution at these different time scales.

#### 4.4.2. Phylogenetic rate analyses

Three-species alignments incorporating the great tit allowed us to examine positive and negative selection in a likelihood framework, testing models of branch-specific rate variation versus a null model of equal rates across the three branches of the tree. Adding a third species left us with 4721 alignments with a minimum length of 300 bp and an average length 1124.7 bases. We tested a null model of equal rates across all three lineages with alternatives in which either violet-eared waxbill or zebra finch was allowed an independent rate. By allowing rate to vary on the zebra finch lineage, we detected significant variation in rate in 347 genes, 112 of which showed deceleration and 235 of which showed acceleration. However, only five of these genes (KIAA1712, DPM1, FOXK2, MXI1 and ATP6V0A) were significantly accelerated after FDR correction and none were significantly decelerated. DPM1 is annotated with a number of intriguing GO functions related to brain and behaviour, such as axon target recognition, axonogenesis, eating behaviour, adult walking behaviour and response to stress. These GO categories were significantly enriched (*p* < 0.05); but in all cases, the observation was one gene relative to an expectation of 0 based on a sample of five accelerated genes. GO analysis of the full list of 235 accelerated genes (without correction for multiple comparisons) revealed enrichment of a number of other functional categories including cAMP-dependent protein kinase complex, regulation of protein amino acid phosphorylation and forebrain development, among others (table 1). These enrichments, however, were non-significant after multiple testing corrections.

**Table 1.** GO analysis of functional over-representation of genes showing significant rate acceleration (*p* < 0.05) in the zebra finch lineage relative to violet-eared waxbill and great tit.

GO	GO description	total	expected	observation	*p*-value	adjusted *p*-value
GO:0005952	cAMP-dependent protein kinase complex	4	0	3	0.00046	0.24
GO:0008603	cAMP-dependent protein kinase regulator activity	5	0	3	0.0011	0.24
GO:0005088	Ras guanyl-nucleotide exchange factor activity	6	0	3	0.0021	0.24
GO:0015386	potassium : hydrogen antiporter activity	2	0	2	0.0024	0.24
GO:0090181	regulation of cholesterol metabolic process	2	0	2	0.0024	0.24
GO:0019933	cAMP-mediated signalling	3	0	2	0.0071	0.24
GO:0017016	Ras GTPase binding	3	0	2	0.0071	0.24
GO:0015385	sodium : hydrogen antiporter activity	3	0	2	0.0071	0.24
GO:0051259	protein oligomerization	9	0	3	0.008	0.24
GO:0001932	regulation of protein phosphorylation	9	0	3	0.008	0.24
GO:0005083	small GTPase regulator activity	11	1	3	0.015	0.24
GO:0031594	neuromuscular junction	5	0	2	0.022	0.24
GO:0017157	regulation of exocytosis	5	0	2	0.022	0.24
GO:0016442	RNA-induced silencing complex	5	0	2	0.022	0.24
GO:0015299	solute : hydrogen antiporter activity	5	0	2	0.022	0.24
GO:0006885	regulation of pH	5	0	2	0.022	0.24
GO:0003729	mRNA binding	13	1	3	0.024	0.24
GO:0051056	regulation of small GTPase-mediated signal transduction	25	1	4	0.033	0.24
GO:0006814	sodium ion transport	15	1	3	0.035	0.24
GO:0016607	nuclear speck	16	1	3	0.042	0.24
GO:0000082	G1/S transition of mitotic cell cycle	7	0	2	0.043	0.24
GO:0000932	cytoplasmic mRNA processing body	7	0	2	0.043	0.24
GO:0045931	positive regulation of mitotic cell cycle	7	0	2	0.043	0.24
GO:0006417	regulation of translation	7	0	2	0.043	0.24
GO:0007265	Ras protein signal transduction	7	0	2	0.043	0.24
GO:0005488	binding	323	16	24	0.044	0.24

In the violet-eared waxbill lineage, we identified significant rate variation in 282 genes, of which 107 were decelerated and 175 accelerated. Only one gene, NCKAP1, was significant after FDR correction (*q* < 0.05). NCKAP1 is associated with five GO terms: apoptotic process, central nervous system development, integral to membrane, lamellipodium membrane and protein binding. The 175 accelerated genes are mildly (but not significantly) enriched for GO terms including neuropeptide receptor activity, mitochondrial respiratory chain complex 1, extracellular ligand-gated ion channel activity, neurotransmitter receptor activity (table 2).

**Table 2.** GO analysis of genes showing significant rate acceleration in the violet-eared waxbill lineage relative to zebra finch and great tit (*p* < 0.05). A number of neruobiologically interesting categories are slightly enriched (Fishers test, *p* < 0.05), but fall short of statistical significance after correction for multiple testing (adjusted *p*-value).

GO	GO description	total	expected	observation	*p*-value	adjusted *p*-value
GO:0005230	extracellular ligand-gated ion channel activity	14	1	4	0.0013	0.13
GO:0006836	neurotransmitter transport	7	0	3	0.0015	0.13
GO:0016620	oxidoreductase activity, acting on the aldehydeor oxo group of donors, NAD or NADP as acceptor	10	0	3	0.0048	0.19
GO:0004222	metalloendopeptidase activity	21	1	4	0.0063	0.19
GO:0004890	GABA-A receptor activity	11	0	3	0.0064	0.19
GO:0007218	neuropeptide signalling pathway	13	0	3	0.01	0.19
GO:0045211	post-synaptic membrane	26	1	4	0.014	0.19
GO:0046854	phosphatidylinositol phosphorylation	15	1	3	0.016	0.19
GO:0005694	chromosome	16	1	3	0.019	0.19
GO:0031625	ubiquitin protein ligase binding	33	1	4	0.031	0.19
GO:0006351	transcription, DNA-dependent	49	2	5	0.032	0.19
GO:0045893	positive regulation of transcription, DNA-dependent	85	3	7	0.035	0.19
GO:0007166	cell surface receptor signalling pathway	20	1	3	0.035	0.19
GO:0016021	integral to membrane	476	17	26	0.037	0.19
GO:0045202	synapse	37	1	4	0.045	0.21
GO:0030054	cell junction	38	1	4	0.048	0.21

## 5. Discussion

We have generated a brain transcriptome from a developing model species for neurobiology and behaviour, the violet-eared waxbill. The brain transcriptome encompasses partial or complete coverage of orthologues to 11 064 zebra finch genes, or 64% of currently annotated zebra finch genes. Our deep sequencing of two full Roche 454 plates reconstructed longer transcripts than any previously produced bird transcriptome, as measured by an N50 score and average transcript length. The combination of normalized libraries, long read lengths and deep sequencing probably explain this success. Despite this, our focus on a single tissue sample (whole brain), and the challenges of detecting and assembling rare transcripts, prevented us from attaining ‘complete’ transcriptome coverage.

As might be predicted in a metabolically costly tissue like the brain, we found significant over-representation of GO terms associated with mitochondrial function and cellular energetics. We also observed a striking lack of expression of OR genes. Although birds were long considered not to have an important sense of smell, the discovery of numerous OR genes [55,56], and evidence for the use of smell [57–59], suggests that olfaction in birds has been underappreciated. In the waxbill brain transcriptome, however, we detected only expression of two ORs out of a large pool of such genes in the genome. While this pattern is striking, ORs are predominantly expressed in the olfactory epithelium [60,61]. Despite normalizing our library to minimize the impact of highly expressed genes, it is possible that OR expression is simply too low in the brain to be detected. While OR repertoires have begun to be characterized across bird lineages, there remains very little information on when, where and at what level these receptors are expressed [62–64]. A prior analysis in the zebra finch also found scarce evidence for olfactory gene expression in the brain [65]. Detailed analyses of ORs in the zebra finch genome also suggest that current Ensembl annotations do not fully describe the OR repertoire [66]. Deeper and tissue-specific RNA sequencing in zebra finches and other passerines, as well as improvements to the genome assembly itself, will improve our understanding of OR repertoires and expression patterns.

We also found poor representation of the immune genome in the waxbill brain. This is consistent with the notion of the brain being ‘immune privileged’ [67,68]. Zebra finch brain ESTs have, however, revealed expression of MHC class I in the brain [8,53,69], and our waxbill transcriptome also includes an MHC class I gene. The finding of MHC class I genes in songbird brains is consistent with neurobiological roles for these genes in mammalian systems [70]. As a whole, however, immune genes are poorly represented in the brain transcriptome.

We have also described patterns of molecular evolution over the last 15 Myr in the estrildid finches. Although previous estimates of avian nucleotide substitution rates were derived from deep evolutionary divergences, we found that our estimate of genome-wide *ω*, 0.13, closely matches those derived in a previous study comparing chicken and zebra finch [17]. This ratio is similar to estimates from *Drosophila* [5] and rodents [71], and is lower than estimates from primates [71]. This supports the hypothesis that birds have had relatively large effective population sizes over their history, resulting in relatively efficient purifying natural selection. The fact that we focused on brain-expressed transcripts, however, also probably biases this estimate downwards. Inclusion of RNAs from gonadal and immune-active tissues might incorporate a disproportionate number of fast-evolving genes, bumping the overall estimate up slightly. Based on our findings, we conclude that the estrildid finch brain transcriptome, at least, has evolved under efficient purifying selection. This pattern differs markedly from the recent observation of high rates of protein evolution among two closely related falcons [12].

We did not find any difference in *ω* between the two focal taxa here, zebra finch and violet-eared waxbill. This was somewhat contrary to our expectation. Zebra finches are colonial, abundant throughout Australia and appear to have had extremely large effective population sizes in their history [62]. Using a set of 30 sequence loci, Balakrishnan & Edwards [72] estimated effective population size (*N*_e_) for Australian zebra finches to be around seven million. Given this large effective population size, we suspected that zebra finches would show relatively efficient purifying selection when compared with violet-eared waxbills. Violet-eared waxbills, however, also have a broad range in southern Africa [73], and population sizes for this species therefore must also be large enough to effectively purge slightly deleterious mutations that would otherwise elevate genomic estimates of *ω* [74]. Large effective population sizes and efficient natural selection may be the norm for passerine bird species. A lingering question is the extent to which domestication of the zebra finch over the last 100 years or more has shaped its genome sequence. The zebra finch genome assembly is based on a captive bird, potentially influencing our estimates of nucleotide substitution relative to that derived from a wild bird.

Our analyses confirmed an increased rate of evolution on the Z sex chromosome. Previous studies of avian genomes have shown this and attributed fast evolution to the lower effective population size of the Z chromosome [16,75]. Despite this fast evolution of the Z chromosome, we did not find strong evidence of reproductive genes (expressed in the brain) being a specific target of positive selection in the genome. We also found a low rate of polymorphism on the Z chromosome, here measured as SNP density. This finding is also consistent with the hypothesis of purifying natural selection acting on the Z sex chromosome [76]. An unexpected outlier in our rate and SNP analyses was chromosome 4A. Chromosome 4A showed the opposite pattern from the Z chromosome in that *ω* was relatively low and SNP density was high. We speculate that this pattern reflects a relaxation of selection on the genes of chromosome 4A, perhaps in association with the fission of chromosome 4, which gave rise to 4A. Chromosome 4A is also of special interest as it has been described as a neo-sex chromosome in warblers (Sylvioidea), with linkage to the Z chromosome [77]. It is not clear whether the unusual patterns of variation and divergence we discovered might be related to the interesting biology of the chromosome.

Strong purifying selection was also evident in our gene-specific analyses of evolutionary rates. In pairwise comparisons, we found few genes with *ω* > 1 and phylogenetic tests for rate acceleration similarly uncovered only a handful of cases of strongly accelerated evolution. In many analyses of positive selection, functional categories of immune response and reproduction are over-represented among positively selected genes [78]. We did not find such a signature in our analysis save for a signature of rapid evolution of cytokine genes. We attribute this primarily to the fact that such genes, those involved in immunity and reproduction, are not well represented in our brain transcriptome. Immune genes were in fact significantly under-represented in our dataset as a whole (figure 2). Analyses of complete avian genomes, however, also failed to detect this common signature [17]. Nam *et al*. [17] attributed this to the fact that in their study, evolutionary comparisons of divergent species diluted the signature of adaptive bouts of evolution. Immune genes, however, have been shown to be evolving rapidly on relatively recent time scales in comparisons of turkey and chicken genomes [9]. Enhancing the waxbill brain transcriptome with genes better represented in other tissue (e.g. spleen and gonads) might help us to test for positive selection in immune- and reproduction-related genes.

Our study used ‘branch’ models to identify genes that show a signature of accelerated evolution across the entire gene. This approach is conservative because often only a few residues within a protein undergo adaptive evolution [69]. We refrained from using more powerful ‘branch site’ models given that our analysis involved only three passerine species for which high-quality transcriptomes were available, giving us limited power to investigate site-specific patterns. As new transcriptomes and whole genomes are sequenced for birds, we will have improved power to detect positive selection and uncover genes underlying traits of interest. The estrildid finches, in particular, represent a promising focal point for studying how genomic evolution is linked to neurobiological and behavioural change.

## Supplementary Material

Gene Ontology analysis of detected trasncripts
